# Natural variation and artificial selection of photoperiodic flowering genes and their applications in crop adaptation

**DOI:** 10.1007/s42994-021-00039-0

**Published:** 2021-06-02

**Authors:** Xiaoya Lin, Chao Fang, Baohui Liu, Fanjiang Kong

**Affiliations:** Innovative Center of Molecular Genetics and Evolution, School of Life Sciences, Guangzhou University, Guangzhou, China

**Keywords:** Adaptation, Photoperiodic flowering, Crops, Artificial selection

## Abstract

Flowering links vegetative growth and reproductive growth and involves the coordination of local environmental cues and plant genetic information. Appropriate timing of floral initiation and maturation in both wild and cultivated plants is important to their fitness and productivity in a given growth environment. The domestication of plants into crops, and later crop expansion and improvement, has often involved selection for early flowering. In this review, we analyze the basic rules for photoperiodic adaptation in several economically important and/or well-researched crop species. The ancestors of rice (*Oryza sativa*), maize (*Zea mays*), soybean (*Glycine max*), and tomato (*Solanum lycopersicum*) are short-day plants whose photosensitivity was reduced or lost during domestication and expansion to high-latitude areas. Wheat (*Triticum aestivum*) and barley (*Hordeum vulgare*) are long-day crops whose photosensitivity is influenced by both latitude and vernalization type. Here, we summarize recent studies about where these crops were domesticated, how they adapted to photoperiodic conditions as their growing area expanded from domestication locations to modern cultivating regions, and how allelic variants of photoperiodic flowering genes were selected during this process. A deeper understanding of photoperiodic flowering in each crop will enable better molecular design and breeding of high-yielding cultivars suited to particular local environments.

**Supplementary Information:**

The online version contains supplementary material available at 10.1007/s42994-021-00039-0.

## Introduction

The history of agriculture is the process of artificial selection of numerous plants and animals. Humans began to settle down and establish agricultural societies in which they artificially domesticated wild animals and cultivated plants as crops. Natural mutations at different gene loci provided the driving force for intensive artificial selection, which can be divided into two stages: domestication and improvement. The domestication stage is characterized by continuous artificial selection by farmers of individual plants with excellent agronomic traits and further expansion of planting. In this process, landraces are developed. During the improvement stage, breeders cross varieties with different desired agronomic traits to obtain cultivars that combine multiple advantages (Meyer and Purugganan 2013). As human societies developed, the spread of different crops has brought a more stable food supply, so that hunter-gatherers could settle down in villages with larger populations and ultimately form more complex societies and cultures. However, plants respond strongly to changes in photoperiod and do not adapt easily to new environments and different latitudinal areas, which has sometimes hindered human agricultural activities.

Flowering plants sense day/night length as a seasonal cue to flower. Plants can be categorized into three groups according to their photoperiodic flowering response. Long-day (LD) plants flower when exposed to light periods longer than a certain critical time; short-day (SD) plants require a long period of darkness, and thus a short day, before they flower; and day-neutral plants flower regardless of the day length (Garner and Allard 1920). This photoperiodic habit of flowering is usually determined by where the plant originates. Plants that originated near the equator, where day length is relatively constant throughout the year, usually do not respond to changes in day length. Plants that originated at low or high latitudes usually flower in response to short or long days, respectively (Brambilla et al. 2017). In addition, some plants require vernalization (a long period of cold) to relieve the inhibition of flowering before they respond to photoperiod signals.

Most plants have an obvious photoperiodic flowering response, and the time of flowering determines the adaptability and influences the final yield of crops. Therefore, photoperiodic flowering is a major factor in the process of artificial selection. In this regard, one of the goals of farmers and breeders is to acclimate crops to local photoperiod conditions, such that they flower and mature in an appropriate season or period, and ultimately meet human needs for crop yield. In this process, some alleles of important photoperiod response genes have been unconsciously selected by humans. Artificial selection thus produces crops that are superior to their ancestors in terms of agronomic traits may better adapt to new areas than their progenitors. Each crop has a unique history of domestication and improvement. In this review, we summarize how six crops, two LD crops and four SD crops, changed to adapt to new photoperiodic conditions over their evolutionary histories.

## Wheat and barley: vernalization affects regional photoperiodic flowering acclimation

Wheat and barley are typical LD crops that originated in western Asia, in and near the so-called Fertile Crescent, an area characterized by the alternation of cold and warm seasons (Jones et al. 2008; Haas et al. 2019). Barley was domesticated from its wild ancestor *Hordeum spontaneum*. The emergence of wheat (*Triticum aestivum*) involved two stages: *Triticum urartu* (with an AA genome) and *Aegilops peltoides* (BB genome) hybridized to form emmer wheat, *Triticum turgidum* (AABB genome), which then hybridized with *Aegilops tauschii* (DD genome) to produce the hexaploid wheat *Triticum aestivum* (AABBDD genome) (Kilian et al. 2010). Wheat and barley can each be characterized into two categories according to their requirement for vernalization: the winter types require a period of cold before they become competent to flower, whereas the spring types do not. Two genes are associated with vernalization in cereals—*VRN1/FUL1/WAP1* and *VRN2*—both of which are critical for adaptation to autumn sowing. *VERNALIZATION1* (*VRN1*) encodes an AP1-like MADS-box transcription factor, VERNALIZATION1, a homolog of *Arabidopsis AP1*, and its transcription is induced by vernalization (Yan et al. 2003). The *VRN2* locus contains two similar genes, *ZCCT1* and *ZCCT2* (Yan et al. 2004). VRN1 binds directly to the promoter regions of *VRN2* (both *ZCCT1* and *ZCCT2*) and *VRN3* (also known as *FT1*) to regulate their transcription (Deng et al. 2015). VRN2 inhibits *FT1* expression, and after prolonged cold exposure, plants can flower under LD conditions.

Although vernalization is not a photoperiodic response, it enables winter sowing in addition to regular spring sowing. The differences in sowing and growing times lead to distinct preferences for photoperiod sensitivity when breeding winter or spring wheat and barley. For example, the spring types of grain crops are sown in spring and harvested in autumn. Their growing season occurs in summer, when crops respond to LD conditions at high latitudes and to SD conditions at low latitudes. At high latitudes, to prevent loss of yield caused by early heading, the response to LD-induced heading must be weaker. To avoid heat and possible summer drought during the seed setting stage in tropical regions, late heading should be discouraged and photosensitivity reduced in cereals through artificial selection. The winter types are sown in autumn and harvested the next summer, so the whole growing season consists of SD conditions. Therefore, reduced sensitivity to SD conditions is required for heading. However, at high latitudes, the summer is mild and conducive to setting, so the heading date can be relatively late compared to that of winter cereals adapted to relatively low latitudes (Table S1).

The major locus conferring photoperiodic sensitivity in cereals is *PHOTOPERIOD1* (*Ppd1*), which belongs to the pseudo-response regulator (PRR) gene family. The corresponding gene in barley is known as *Ppd-H1* because barley contains the HH genome (Turner et al. 2005). The prevalence of the *Ppd-H1a* allele (the insensitive allele, Table S1), which originated from wild barley, increased as barley adapted to the high latitudes of Europe. This indicates that as barley was acclimating to high latitudes with LD conditions, people chose late-heading cultivars to ensure sufficient yield (Jones et al. 2008). The corresponding genes in wheat are *Ppd-A1* (in the AA genome), *Ppd-B1* (in the BB genome), and *Ppd-D1* (in the DD genome); *Ppd-D1* contributes the most to the heading date of winter wheat. The two alleles of this gene, *Ppd-D1a* (which is missing 2,089 bp in the 5′ upstream region of *PPD-D1*) and *Ppd-D1b*, can lead to a difference of 10 days in the heading time (Würschum et al. 2018; Seki et al. 2011). *Ppd-D1a* is favored in winter wheat in the southern parts of both Europe and Japan to accelerate heading and thereby avoid hot summers or local rainy seasons (Würschum et al. 2018; Seki et al. 2011). In Chinese wheat, the frequency of the *Ppd-D1a* allele increased from 38.6% in the landrace to 90.6% in improved cultivars, indicative of significant selection for this allele during the improvement process (Yang et al. 2009). In the northern United States, spring wheat is grown in advance to increase the duration of vegetative growth, thus increasing yields. With this strategy, the photoperiod-sensitive allele *Ppd-D1b* is preferable (Lanning et al. 2012).

The effect of *Ppd-B1* on wheat photoperiod flowering is mainly reflected in its copy number variation. The copy number of *Ppd-B1* increases from north to south in European winter wheat cultivars. Higher *Ppd-B1* copy number can cause the induction of gene expression resulting in photoperiod insensitivity, enabling a cultivar planted in a southern area to head early and avoid high temperatures and drought (Würschum et al. 2015, 2018; Díaz et al. 2012). Mutations in the *Ppd-B1* gene region can also lead to changes in gene function. In Japanese wheat, a 308-bp insertion upstream of *Ppd-B1* can cause photoperiod insensitivity and an early flowering phenotype (Nishida et al. 2013b). The effects of *Ppd-D1* and *Ppd-B1* on photoperiod flowering are additive: an increase in the copy number of *Ppd-B1* leading to earlier heading is observed in both the *Ppd-D1b* and the photoperiod-insensitive *Ppd-D1a* backgrounds (Würschum et al. 2018).

In winter wheat in Japan, heading date is significantly earlier in the *Ppd-B1a*/*Ppd-D1a* genotype than the *Ppd-B1b*/*Ppd-D1a* genotype, and winter wheat with *Ppd-B1a* is distributed in mid- and low-latitude regions (Seki et al. 2011). In addition, the *Ppd-B1a*/*Ppd-D1b* genotype is not found in improved cultivars of Japanese wheat, suggesting that the mutations of *Ppd-D1a* and *Ppd-B1a* are part of a stepwise selection process that finely regulates heading time in regions with different climates (Seki et al. 2011).

There is a similar insensitive mutant for *Ppd-A1*: a 1,085-bp deletion upstream of *Ppd-A1a* in Japanese wheat can lead to photoperiod-insensitive phenotypes with early flowering (Nishida et al. 2013b). Among 240 Japanese cultivars, only a few Hokkaido winter wheat cultivars, and no spring wheat cultivars, carry the *Ppd-A1a* allele. Cultivars with the *Ppd-A1a* allele show early heading when grown in the Kanto region, but early heading by only 2.5 days in the Hokkaido region. The mutant allele of this gene does not seem to be widespread, however (Seki et al. 2013). Perhaps the presence of *Ppd-A1a* further fine-tunes the photoperiod sensitivity of winter wheat in relatively high-latitude areas.

*PhyC* and *CO* are also important for the photoperiod flowering of barley and wheat, but whether they play a significant role in latitude adaptability is not known (Beales et al. 2005; Chen et al. 2014; Nishida et al. 2013a; Shaw et al. 2020; Mulki and von Korff 2016).

## Soybean: two directions of evolution and adaptation -- promote or delay flowering

Soybean (*Glycine max*) was most likely domesticated in central China around the Huang–Huai Valley, a mid-latitude temperate region, from its wild progenitor *Glycine soja* 5,000 years ago (Sedivy et al. 2017). The subsequent worldwide expansion of soybean to South Korea, Japan, South Asia, and Southeast Asia began 2,000 years ago, and the species reached North America in 1765 and South America in the early twentieth century (Stacey 2008). Adjustment of flowering phenology to photoperiod-based seasonal cues in particular growth environments may have been a driving force for the expansion of cultivation areas toward higher and lower latitudes. To adapt to the northern parts of China, Korea, and Japan, soybean required reduced photoperiod sensitivity and early flowering to allow it to mature before the first frost. However, to better suit lower-latitude areas such as Brazil and Southeast Asia, flowering needed to be delayed to maximize yield.

Natural variations at soybean photoperiodic flowering gene loci contributed to soybean domestication and subsequent diversification. In the process of domestication, mutations in *Tof12* (*PRR3a*) laid the foundation for adaptation to primitive cultivation of soybean and further adaptation towards northern areas (Li et al. 2019,2020a; Wang et al. 2020; Lu et al. 2020). The loss-of-function allele *tof12*, which results in early flowering, is enriched in landraces compared to wild soybean, especially in the northeast region of China (Lu et al. 2020). Recently, for the first time, early flowering phenology was molecularly confirmed as a core domesticated trait in soybean (Gong 2020). It is easy to imagine early flowering as a trait selected during domestication because modern agriculture favors early and synchronized maturation. However, *GsFT2c* in *G. soja* is a functional gene, unlike *GmFT2c* in *G. max*, and overexpression of *GsFT2c* in *Arabidopsis* can induce early flowering (Wu et al. 2017). A variant of *GmFT2c* with a transposon insertion mutation allele is basically fixed in soybean landraces, indicating that this mutation occurred in the early stages of domestication. Hence, soybean *FT2c* may have been involved in the evolution of the regulation of photoperiodic flowering during soybean domestication (Li et al. 2014; Wu et al. 2017). Although domesticated *GmFT2c* shows no obvious latitudinal trend in distribution, it causes late flowering in soybean only under SD conditions. Mutation of *FT2c* might thus be classified as an early step of adaptation to lower latitudes, which does not influence adaptation to higher latitudes.

As plants acclimated to higher latitudes in Asia and North America, various mutations affecting reproductive phenology were selected for and contributed to the expansion of cultivation areas in the process of diversification or improvement. The loss-of-function *tof11* (*prr3b*) and *e2* (*gi*) alleles contribute to flowering time adaptation in which earlier flowering and maturation is preferred (Wang et al. 2016; Lu et al. 2020; Xu et al. 2013; Watanabe et al. 2011). E3 and E4 are two phytochrome A proteins that predominate under high red/far-red (R/FR) ratio light and low R/FR ratio light, respectively (Cober et al. 1996a, b; Liu et al. 2008; Watanabe et al. 2009). E1 and E1La/b, the legume-specific B3 domain transcriptional factors, inhibit the transcription of florigen, *GmFT2a*, and *GmFT5a* (Xia et al. 2012; Xu et al. 2015; Zhu et al. 2019). Different combinations of natural variations at the *E3*, *E4*, and *E1*/*E1lb* loci, such as *e3 e4*, *e1 e3*; *e1 e4*, *e1-as e3*; and *e1-as e1lb e3*, reduce soybean photoperiod sensitivity, expanding the cultivation area of soybean toward higher latitudes (Xu et al. 2013; Zhu et al. 2019). The *E3*, *E4*, and *E1* family may have complicated genetic interactions in which *E1* plays the central role in the photoperiodic flowering signaling pathway (Xu et al. 2013; Xia et al. 2012; Zhu et al. 2019). In addition, the soybean SKIP ortholog GmGBP1 positively functions in photoperiod-mediated flowering pathways. The promoter region of GmGBP1 is under selection during soybean migration to higher latitudes (Zhao et al. 2018).

Mutation of the *J* (*ELF3* homologue) locus, on the other hand, provides advantages for soybeans in adapting to lower latitudes and increases yield (Lu et al. 2017). The utilization of the various *j* alleles has made Brazil the second-largest soybean producer in the world and changed the global soybean trade pattern. Loss of function of *j* releases the inhibition of *E1* transcription, which leads to late flowering. Since *J* is genetically dependent on *E1* (Lu et al. 2017), the dominant *E1* allele might be the basis for adaptation to low latitudes (Lin et al. 2020; Miranda et al. 2020). More evidence may be needed to support such a conclusion.

Soybean has 10 *FT* homologues, of which *GmFT2a* and *GmFT5a* play the major roles in promoting flowering (Kong et al. 2010). Polymorphism association analysis also showed that *GmFT2a* and *GmFT5a* induce flowering time in a panel of 127 accessions (Jiang et al. 2019). Haplotypes of *GmFT2a* and *GmFT5a* display varying frequency among different soybean maturity groups (a classification based on flowering time according to the latitudinal adaptation), implying that they are involved in the adaptability of soybean to tropical and high-latitude regions, respectively (Zhao et al. 2016; Cai et al. 2020).

## Rice: reduce and maintain photoperiod sensitivity in the north and south, respectively

Rice is one of the world’s most important staple foods. Cultivated rice (*Oryza sativa*) is domesticated from its progenitor (*Oryza rufipogon*). There are several subspecies of rice, including *japonica* and *indica*, and there has long been debate about their origins and domestication locations. Some genetic studies have shown that the *japonica* subspecies was first domesticated in the Yangtze River valley (Molina et al. 2011) or the Pearl River Basin (Huang et al. 2012) in China, while archeological evidence suggests that rice was first domesticated in the Yangtze River valley in southern China 10,000–8,000 years ago (Gross and Zhao 2014). Some researchers believe that *japonica* was domesticated first and then spread north, with one branch spreading south and entering southeast Asia, where it crossed with local wild rice and underwent a second domestication, resulting in a new subspecies, *indica* (Huang et al. 2012). There is also evidence suggesting that these two subspecies were independently domesticated (Civáň et al. 2015; Wang et al. 2018).

Rice is widely cultivated between latitudes 53° N and 40° S. As rice has adapted to higher latitudes, its sensitivity to photoperiod has been gradually lost to avoid delayed flowering in LD conditions. Among rice varieties grown in extreme northern regions, including northern Europe, northern Japan, northeastern provinces of China, and the far eastern parts of Russia (40°–53° N), all elite cultivars have no or very weak photoperiod response (Fujino and Sekiguchi 2005; Wei et al. 2008). Several genes, including *Hd1*, *Ghd7*, *Ghd8*, *Hd2*, *DTH2*, *Hd6*, and *Hd16*, play pivotal roles in reducing rice photosensitivity in LD conditions.

*Hd1* is a homologue of the *Arabidopsis* gene *CONSTANS* (Yano et al. 2000). Hd1 has a dual role in rice photoperiodic flowering: It inhibits flowering under LD conditions but promotes it under SD conditions (Yano et al. 2000; Hayama et al. 2003). The mutant form *hd1* is widely selected in high latitudes and is also found in tropical regions (Takahashi et al. 2009; Goretti et al. 2017). A 2-bp deletion in *Hd1* appears in many subspecies, which implies that this mutation occurred and was selected for before these subspecies differentiated (Itoh et al. 2018; Fujino et al. 2010). Ghd7/Hd4 is a transcription factor containing a CCT domain but not a B-box zinc finger structure, and it does not have high homology with other CCT proteins of *Arabidopsis*, rice, wheat, or barley. The distribution of its haplotype in Asia varies significantly with latitude and its corresponding duration of growth. Rice that possesses the loss-of-function allele of *ghd7* has reduced photoperiod sensitivity (Xue et al. 2008; Gao et al. 2014). Hd5/Ghd8/DTH8/LHD1 is a putative HAP3/NF-YB/CBF-A subunit of the CCAAT-box-binding transcription factor, which causes late heading. Rice with a mutant form of *ghd8* has a decreased sensitivity to photoperiod (Dai et al. 2012; Fujino et al. 2013; Wei et al. 2010).

The three genes *Ghd7*, *DTH8/Ghd8*, and *Hd1* combine to synergistically and finely regulate the flowering time of rice, which contributes greatly to rice’s adaptability to different latitudes and weather conditions. In the absence of Ghd7 and Ghd8, Hd1 is a heading-promoting factor; when Ghd7 and Ghd8 are present, Hd1 can form a complex with either or both to inhibit the expression of *Ehd1*, thereby inhibiting the transcription of *Heading date 3a* (*Hd3a*) and *Rice flowering locus T1* (*RFT1*) (Zong et al. 2020). Allelic combinations of these three genes largely determine the photoperiodic response of rice. When all three proteins are present, rice shows strong or very strong photoperiod sensitivity; if any of the three proteins is absent, rice exhibits moderate photoperiod sensitivity; and if two or three are missing, rice has weak photoperiod sensitivity (Zong et al. 2020). In tropical and subtropical regions of China, almost all wild rice accessions and landraces have strong or very strong photoperiod sensitivity and grow quite poorly at other latitudes. In central and southern China, as an adaptation to the double-season cropping system, early-maturing varieties predominate. As a result, *indica* cultivars (with the genotype *hd1 Ghd7 ghd8*), which have weak photoperiod sensitivity, are generally cultivated there. In central China, where single cropping is used, the heading time of rice can be relatively delayed to enhance yield. There, *japonica* cultivars (with the genotype *Hd1 Ghd7 Ghd8*), which have strong photoperiod sensitivity, are cultivated. In the northernmost part of China, rice types with weak photoperiod sensitivity are generally cultivated, including ecotypes with mutations in all three genes (Zhang et al. 2015; Zong et al. 2020; Xue et al. 2008). However, it has also been suggested that functional Ghd8 is crucial for rice to confer cold tolerance (Wang et al. 2019). As a result, most cultivars at high latitude would carry Ghd8 (Zhang et al. 2015; Li et al. 2015).

Hd2/DTH7/PRR37 (Pseudo-response 37) is a member of the circadian clock, and its mutant form is distributed in both high and low latitudes (Koo et al. 2013). PRR37 is a heading inhibitor under natural LD conditions, while the flowering effect is alternative under natural SD conditions depending on the effects of other genes (Zhang et al. 2019). Cultivars with *hd2/prr37* and *hd4/ghd7* double mutation are distributed in the northernmost regions. These two mutations are correlated with cumulative temperature (Koo et al. 2013; Li et al. 2015).

Wu et al. (2013) found that *Days to heading on chromosome 2* (*DTH2*) genes, encoding proteins in the CONSTANS-like (COL) family, can induce the expression of *Hd3a* and *RFT1*. Two kinds of functional nucleotide polymorphisms can lead to early flowering and improve the fitness of rice in high latitudes (Wu et al. 2013).

RFT1 and Hd3a are both florigens of rice. RFT1 dominates under LD conditions, while Hd3a plays a role mainly under SD conditions (Komiya et al. 2008; Tsuji et al. 2011). Functional *RFT1* is required for rice to adapt to high latitudes. *Indica* and *japonica* cultivated north of 33° N carry functional *RFT1* (Zhao et al. 2015), while wild or cultivated rice strains adapted to low latitudes sometimes carry *rft1* mutations (Zhao et al. 2015; Ogiso-Tanaka et al. 2013; Naranjo et al. 2014). This is probably because all the above-mentioned genes in rice are genetically dependent on *RFT1* to promote flowering under LD conditions in rice.

*Hd6*, which encodes a subunit of casein kinase 2 (CK2), has kinase activity and modifies Hd1 under LD conditions (Takahashi et al. 2001; Ogiso et al. 2010). Hd16 encodes a casein kinase I (CKI) that functions to phosphorylate Ghd7. A nonsynonymous substitution in *Hd16* results in decreased kinase activity and reduced photoperiodic sensitivity of rice (Hori et al. 2013; Kwon et al. 2014). Hd16 also enhances the function of Hd1 under both LD and SD conditions (Nemoto et al. 2018). Breeders directionally selected the mutated form of *hd6* and *hd16* in temperate *japonica* to acclimate it to northern regions (Nemoto et al. 2018).

When rice expands to lower latitudes, rice flowering is promoted as the day length becomes shorter. However, in rice, in contrast to soybean, delayed flowering time was not strongly selected for at lower latitudes to increase yield. The mutated form of *hd1* was selected to avoid extremely early heading under SD conditions in rice (Kim et al. 2018). Another gene, *Ehd1*, was also edited to avoid extremely early heading under SD conditions (Wu et al. 2020). Rice has a relatively long flowering time (usually over 70 days, according to https://www.ricedata.cn/variety/index.htm) under SD conditions, so local farmers prefer double or triple cropping in the course of a year, rather than prolonging one growth cycle, as the means to increase yield.

## Maize: ambitious to adapt to higher latitudes

Maize (*Zea mays*) was domesticated from teosinte (*Zea mays ssp. Parviglumis*), which originated in the tropical balsas river basin of southwestern Mexico and then spread to high latitudes (Matsuoka et al. 2002). Its range was expanded during pre-Columbian times to include cold temperate regions. Globally, maize is the most widely grown crop (http://faostat.fao.org/); the main producing countries are the United States, China, Brazil, India, and Argentina, with latitudes ranging from 40° S to 45° N (Buckler et al. 2009).

In the early domestication of maize, the early flowering allele of *ZEA CENTRORADIALIS8* (*ZCN8*), an *FT* homologue, was strongly selected (Lazakis et al. 2011). The expression of *ZCN8* is regulated by the variant SNP-1245, located in a cis-acting element, which causes early flowering by influencing the binding of ZmMADS1 to the promoter region of *ZCN8* (Guo et al. 2018). ZmMADS1 is a transcription factor that affects the flowering time of maize (Alter et al. 2016). After the early flowering allele at SNP-1245 became fixed, the early flowering allele at another variant in the ZCN8 promoter, InDel-2339, was further selected during the spread of maize from tropical to temperate regions; thus, *ZCN8* has an important effect on the latitude adaptability of maize (Guo et al. 2018). A series of mutations in other genes contributed to the acclimation of maize to higher latitudes in North America, including *ZmCCT9*, *ZmCCT10*, *Vgt1*, *ZmMADS69*, and *ZmMADS67*.

*ZmCCT9* and *ZmCCT10* are homologues of rice *Ghd7* and are the key genes for maize photoperiodic flowering, delaying flowering under LD conditions (Yang et al. 2013; Huang et al. 2018). ZmCCT9/ZmCCT10 negatively regulates the expression of *ZCN8* to inhibit maize flowering under LD conditions (Yang et al. 2013; Hung et al. 2012; Huang et al. 2018). The insertion of a CATCA-like transposable element (TE) in the promoter region of *ZmCCT10* affects the degree of methylation of the promoter sequence, resulting in a decrease in gene expression and earlier flowering. This insertion, which has been selected during the spread from tropical to temperate regions, improves maize latitudinal adaptability (Yang et al. 2013; Hung et al. 2012). In addition, the insertion of a Harbinger-like TE at 57 kb upstream of *ZmCCT9*, which inhibits the expression of *ZmCCT9* through cis-acting, results in early flowering (Huang et al. 2018; Guo et al. 2018). The insertion in *ZmCCT9* accelerated the spread of maize to high-latitude areas (Huang et al. 2018). The CATCA-like TE of *ZmCCT10* and the Harbinger-like TE of *ZmCCT9* are not observed in surveyed teosinte accessions, indicating that these insertions occurred after the initial domestication of maize (Huang et al. 2018; Yang et al. 2013). The TE insertion at *ZmCCT9* shows low frequency at low latitudes but relatively higher frequency at high latitudes. The two insertions at *ZmCCT10* and *ZmCCT9* occurred sequentially following domestication and exhibit different distribution patterns at different latitudes (Huang et al. 2018).

Vgt1/ZmRap2.7 is an AP2/ERF transcription factor that inhibits *ZCN8* expression under LD conditions. Insertion of a miniature inverted-repeat TE (MITE) in *Vgt1* was a major target for selection during the adaptation of maize to cool temperate and high-latitude regions (Salvi et al. 2007; Ducrocq et al. 2008; Romero Navarro et al. 2017).

ZmMADS69/qDTA3-2 is a transcription factor that was cloned by quantitative trait locus (QTL) mapping in offspring of crosses between wild and cultivated species. It inhibits the expression of *Vgt1*, thereby promoting *ZCN8* expression and inducing flowering under both LD and SD conditions. The promoter region of *ZmMADS69* was selected such that temperate maize varieties have higher expression than tropical maize varieties. *ZmMADS69* was a target of artificial selection and played an important role in the spread of maize from the tropics to temperate zones (Liang et al. 2019).

ZCN8 interacts with the basic leucine zipper transcription factor DLF1 (Delayed flowering 1). Through QTL mapping of recombinant inbred lines of maize and teosinte, a QTL *qLB7-1* that affects flowering time was identified. The maize allele flowers early, and *DLF1* is the most likely candidate gene for this locus. DLF1 promotes floral transition by directly binding to the promoter regions of *ZmMADS4* and *ZmMADS67* and activating them in the shoot apex. In the evolutionary process from teosinte to maize, both *DLF1* and *ZmMADS67* were under selection and underwent decreases in nucleotide diversity, whereas *ZmMADS4* was not under selection. DLF1 and ZmMADS67 may also play an important role in maize adaptation to higher latitudes (Sun et al. 2020).

Besides the above-mentioned genes, investigation of a nested association mapping population also revealed many QTLs that associate with flowering time and adaption, such as *DWARF8*, *ZmPRR37*, *ZmPHYC2*, and *ZmCCA1* (Romero Navarro et al. 2017; Buckler et al. 2009; Li et al. 2016). There is some genetic evidence that they control maize flowering time (Li et al. 2020b; Thornsberry et al. 2001). Allelic geographical distributions may help clarify how these genes changed to adapt to different latitudes in evolutionary history. In contrast to the gradually improving understanding of the adaptation mechanism of maize to North America, little is known about how maize adapted to South America before pre-Colombian times.

## Tomato: from SD plant to day-neutral plant

The origin of tomato (*Solanum lycopersicum*) has long been controversial, but the current consensus is that it was domesticated in the Andean region of South America and Mesoamerica, introduced to Europe by the Spanish in the sixteenth century, and then spread all over the world (Blanca et al. 2012). The most likely ancestral progenitor is *Solanum pimpinellifolium* (Blanca et al. 2012). Cherry tomato is commonly thought to be the intermediate landrace between wild and elite cultivars of tomato (Bergougnoux 2014).

*SP5G* (*SELF PRUNING 5G*) is an *FT* homolog that inhibits flowering. In *S. pimpinellifolium*, it is highly expressed in LD conditions but barely expressed in SD conditions. Reduction of *SP5G* expression or mutation of *SP5G* in improved cultivars attenuates the inhibitory effect of *SP5G* on *SFT* (*SINGLE FLOWER TRUSS*), a florigen that directly induces flowering in tomato, under LD conditions (Shalit et al. 2009). As a result, the sensitivity to photoperiod decreases in LD conditions (Soyk et al. 2017; Pnueli et al. 1998; Zhang et al. 2018).

FTL1 (Flowering Locus T-Like 1) is also an FT homolog but is a promoter of flowering. The expression of FTL1 is much higher under SD conditions than under LD conditions and shows a stronger oscillation pattern. This gene induces the expression of *SFT* under SD conditions. In the *ftl1* mutant, the transcription of *SFT* drops sharply under SD conditions, leading to delayed flowering, which weakens the induction of flowering under SD conditions in tomatoes (Song et al. 2020).

Mutant *ftl1* and *sp5g* genotypes of cherry tomato are distributed near tomato origins, and different allelic combinations are enriched in cherry tomato in the same region, indicating that they were selected independently during domestication (Song et al. 2020). *sp5g* and *ftl1* mutant alleles show a high prevalence in cherry tomato (the landrace). Some wild types carry *sp5g* alleles, but no wild type carries *ftl1* alleles, which implies that *sp5g* was selected before *ftl1* during the tomato domestication process (Song et al. 2020). SP5G inhibits *SFT* in LD conditions, while FTL1 induces *SFT* in SD conditions. The selection of *sp5g* weakens LD photoperiod sensitivity, whereas the enrichment of *ftl1* abolishes the induction of flowering by SD conditions, thus changing tomato from an SD plant to a day-neutral plant (Song et al. 2020).

As tomato migrated from its origin to other parts of Central America and finally was brought to Europe by the Spanish, its circadian rhythms also changed in response to differences in the photoperiods to which it was exposed. Compared with that of wild tomato, the circadian rhythm of improved cultivars has a delayed phase and a longer period, which allowed cultivated tomato to adapt to higher-latitude areas. Genes responsible for altering the circadian rhythm were mutated and fixed during domestication (Müller et al. 2016, 2018). The loss of a single amino acid in EID1 causes delayed phase, prolonged flowering, and elevated chlorophyll content. The *EID1* genome region underwent positive selection during domestication, indicating that humans actively selected phase-delayed mutation to adapt to the longer summer environment (Müller et al. 2016). The *LNK2* gene controls the length of the period. The mutated versions of this gene are not observed in the wild ancestor and are seldom observed in samples before tomato was cultivated in Mexico. *lnk2* was not fixed until the tomato entered Europe in the sixteenth century during its domestication (Müller et al. 2018). The effects of these two genes in regulating the phase and the periodic circadian clock led to synchronization of the circadian rhythm of the internal and external environment as the tomato migrated northward from the equator.

As a response to photoperiodic flowering, cultivated *EID1* delays flowering in both LD and SD conditions; the effect of *lnk2* mutation on flowering is unclear. The functional relationship between the genes *EID1*, *LNK2*, *FTL1*, and *SP5G* in regulating flowering is still a mystery.

## Perspective

**Fig. 1:**
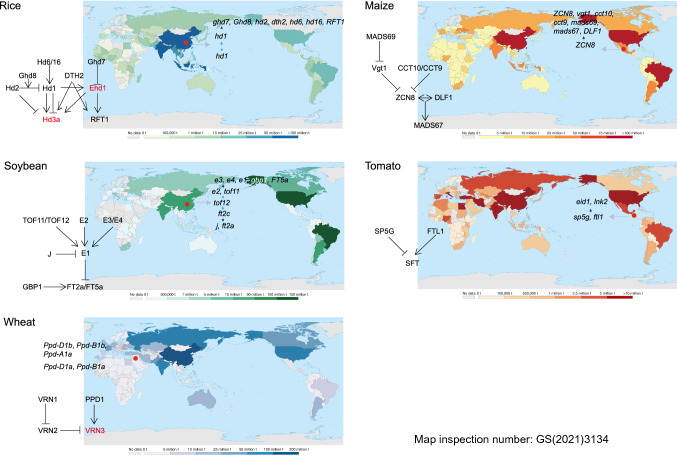
The domestication location, modern cultivation region, and selected photoperiodic flowering genes during latitudinal adaptation of each crop. The world crop production maps for year 2018 were downloaded from https://ourworldindata.org/agricultural-production#cereals and adapted. The domestication sites are indicated by red dots. Genes believed to have been selected during the domestication process are followed by the grey arrows. Genes thought to have been selected after domestication or in the improvement process are followed by blue arrows (with light blue arrows indicating earlier selection than dark blue arrows)

The strategies through which LD crops and SD crops have adapted to different latitudes are varied. As SD plants migrate to high latitudes, flowering and maturity are delayed, which makes it difficult to harvest before winter. It is thus necessary to reduce the photoperiod sensitivity to advance flowering and maturity time. As SD plants adapt to lower latitudes, a balance must be struck between advancing flowering and maximizing yield. When temperate-origin LD plants are cultivated at low latitudes, although the flowering time is delayed and the yield is high, the flowering time is often restricted by the local environment or cropping practices. If the summer is particularly hot or dry, the setting stage should be advanced. In addition, land is usually used for multiple crops in a year in tropical and subtropical regions, so delaying flowering time to maximize yield is not the only option; sensitivity to photoperiod should also be reduced. At high latitudes, reduced response to LD induction of flowering is required to keep yield in an appropriate range (Fig. 1).

Each crop regulates photoperiod in a unique way, but there are some common trends. High-quality crop varieties are those that adapt to various growth conditions in different regions, including diverse photoperiod conditions. The abundant natural variations of photoperiodic flowering genes in crops provide alternatives for farmers and breeders, allowing the selection of cultivars to produce sufficient yields in different photoperiod conditions. By comparing how these crops adapted to various regions, we found that the location of domestication, original flowering day, and contingency in adaptation processes all affected the strategy of adaptation. For example, rice and soybean adopted distinct tactics when they expanded their territories to lower latitudes because rice had a relatively longer original flowering time; when maize expanded to high-latitude areas in the north and south, different combinations of genes were selected in the two cases. It is difficult to discern any uniform trends in these processes. Different target genes were often selected as separate species adapted to the same latitude or area and sometimes even as one species adapted to different areas at similar latitudes (Table 1; Fig. 1). However, certain allelic resources were repeatedly utilized in crop latitudinal adaptation, such as genes in the *PRR* and *FT* families.

**Table 1:** Selected photoperiodic flowering genes during latitudinal adaptation of each crop

Species	Gene names	Encoded proteins	Biological or molecular functions	Corresponding references
Wheat	*Ppd-A1*	PRR	Circadian clock gene	Seki et al. (2011)
	*Ppd-B1*	PRR	Circadian clock gene	Díaz et al. (2012)
	*Ppd-D1*	PRR	Circadian clock gene	Beales et al. (2007)
Barley	*Ppd-H1*	PRR	Circadian clock gene	Turner et al. (2005)
Soybean	*E3*	phyA	Photoreceptor	Watanabe et al. (2009)
	*E4*	phyA	Photoreceptor	Liu et al. (2008)
	*E1*	B3-domain protein	Transcriptional factor	Xia et al. (2012)
	*E2*	GI		Watanabe et al. (2011)
	*GBP1*	SKIP	Transcription cofactor	Zhao et al. (2018)
	*FT5a*		Florigen	Kong et al. (2010)
	*FT2a*		Florigen	Kong et al. (2010)
	*Tof11*	PRR	Circadian clock gene	Lu et al. (2020)
	*Tof12*	PRR	Circadian clock gene	Lu et al. (2020)
	*J*	ELF3	Circadian clock gene	Lu et al. (2017)
	*FT2c*	FT homolog	Florigen	Wu et al. (2017)
Rice	*Ghd7*	CCT domain protein	Transcriptional factor	Xue et al. (2008)
	*Ghd8*	putative HAP3/NF-YB/CBF-A subunit	A subunit of transcription factor	Dai et al. (2012)
	*Hd2*	PRR37	Circadian clock gene	Koo et al. (2013)
	*DTH2*	CONSTANS-like		Wu et al. (2013)
	*Hd6*	a subunit of CK2	Protein kinase	Takahashi et al. (2001)
	*Hd16*	CKI	Protein kinase	Hori et al. (2013)
	*RFT1*	FT homolog	Florigen	Komiya et al. (2008)
	*Hd1*	CONSTANS		Yano et al. (2000)
Maize	*ZCN8*		Florigen	Guo et al. (2018)
	*Vgt1*	AP2/ERF	Transcriptional factor	Salvi et al. (2007)
	*CCT10*	CCT domain protein	Transcriptional factor	Hung et al. (2012)
	*CCT9*	CCT domain protein	Transcriptional factor	Huang et al. (2018)
	*MADS69*	MADS-box	Transcriptional factor	Liang et al. (2019)
	*MADS67*	FUL-like protein	Transcriptional factor	Sun et al. (2020)
	*DLF1*	Basic leucine zipper protein	Transcriptional factor	Sun et al. (2020)
Tomato	*SP5G*	FT homolog		Soyk et al. (2017)
	*FTL1*	FT homolog		Song et al. (2020)
	*EID1*	EID1	F-box protein	Müller et al. (2016)
	*LNK2*	LNK2	Circadian clock gene	Müller et al. (2018)

During domestication and improvement, crops evolve from wild types to landraces and ultimately to improved cultivars. In most crops, these processes are accompanied by some reduction of photoperiod sensitivity. There is an innate contradiction between early maturity and high yield. The best flowering time for a particular crop is the flowering time that makes the best use of the local growth period, thereby maximizing yield. Although day-neutral crops are economical in breeding, and can be cultivated at various latitudes and under most photoperiod conditions, they may not be able to adapt to the natural conditions in a particular area to the greatest extent. Therefore, although a strong photoperiod response is not conducive for adaptation to a wide range of cultivation areas, a certain degree of photoperiod sensitivity should be retained during breeding to make it possible to take advantage of this pivotal physiological factor to increase yields as a crop adapts to different latitudes.

The photoperiod sensitivity of crops is a double-edged sword. Cultivars with lower photoperiod sensitivity have economic benefits and are amenable to cultivation in broad areas but may not produce the greatest yield in every location. As crops become insensitive to photoperiod change through domestication and improvement, the mutant forms of genes selected are usually those upstream of the photoperiodic flowering pathway. Therefore, another breeding method is to choose a cultivar with generally good agronomic traits and then use molecular design to make it completely insensitive to photoperiod. Then, by manipulating the expression of downstream genes such as *FT* homologues, it is possible to create a series of totally photoperiod-insensitive lines with various flowering times. By investigating the planting conditions in certain areas, we can choose the varieties most suitable for the local photoperiod conditions.

Thanks to research on the origins, distribution, and molecular evolution of several crops, it is becoming clear how these crops immigrated to their modern cultivating region from their domestication centers in view of photoperiodic adaptability. With a richer and more detailed understanding of the photoperiodic flowering pathway in each crop, breeders will be fully equipped to molecularly design cultivars according to local environmental and photoperiodic conditions to maximize yield potential.

## Supplementary Information

Below is the link to the electronic supplementary material.

Supplementary file1 (DOCX 23 kb)
