# Supplementary material for: Natural variation and artificial selection of photoperiodic flowering genes and their applications in crop adaptation
Source: aBIOTECH. 2021 Jun 2;2(2):156–69. doi: 10.1007/s42994-021-00039-0 (PMC9590489; doi:10.1007/s42994-021-00039-0)
Supplement: Supplementary file 1 — Supplementary file1 (DOCX 23 kb) [file 42994_2021_39_MOESM1_ESM.docx]

Table S1: Vernalization requirements determine the growing season of wheat and barley and hence their response to photoperiod.

| Direction of expansion | Vernalization type | Photoperiod condition of growing season | Allele selected |
| --- | --- | --- | --- |
| High latitude | Winter type | SD | b (sensitive) |
|  | Spring type | LD | b (sensitive) |
| Low latitude | Winter type | SD | a (insensitive) |
|  | Spring type | SD | a (insensitive) |
